# Associations between humiliation, shame, self-harm and suicidality among adolescents and young adults: A systematic review

**DOI:** 10.1371/journal.pone.0292691

**Published:** 2024-02-08

**Authors:** Anvar Sadath, Katerina Kavalidou, Elaine McMahon, Kevin Malone, Aoibheann McLoughlin

**Affiliations:** 1 School of Public Health, University College Cork, Cork, Ireland; 2 National Suicide Research Foundation, University College Cork, Cork, Ireland; 3 National Clinical Programme, Health Service Executive (HSE), Dublin, Ireland; 4 Department of Psychiatry and Mental Health Research, St. Vincent’s University Hospital, University College Dublin, Dublin, Ireland; University of Rome La Sapienza: Universita degli Studi di Roma La Sapienza, ITALY

## Abstract

**Background:**

Suicide is the second leading cause of death among young people worldwide. Research indicates that negative social contexts involving familial and peer relationships have far-reaching influences on levels of suicidality in later life. While previous systematic reviews have focused on evaluating associations between negative life events such as abuse and bullying in childhood and subsequent suicidality, this systematic review examines the prevalence of, and association between the processes of humiliation and shame in later self-harm, suicidal ideation, and suicide among adolescents and young adults.

**Methods:**

A systematic literature search of databases including MEDLINE, Web of Science Core Collection, CINAHL, PsycINFO, and Embase was conducted to identify potential studies. ProQuest was searched to identify relevant grey literature research. A combination of MESH terms and keywords was used. All original quantitative studies published in English that examined the prevalence, or association between humiliation or shame and suicidal behaviours and/or death by suicide were included. Studies were assessed for methodological quality using Joanna Briggs Institute critical appraisal tools. The protocol was registered with the International Prospective Register of Systematic Reviews (PROSPERO) [CRD42022289843].

**Results:**

Narrative synthesis was performed. A total of 33 studies reporting the prevalence of, or association between humiliation (n = 10) or shame (n = 23) and suicidal thoughts/behaviours were included. The prevalence of humiliation among those with any suicidality ranged from 18% to 28.1%, excluding an outlier (67.1%), with two studies presenting a significant association between humiliation and self-harm in their fully adjusted analyses. The studies that outlined humiliation and suicidal thinking (intent/suicide plan) had no association after adjustment for confounders. For shame, half of the studies found an association in adjusted models (n = 10), and this was evident for both suicidal ideation and self-harm.

**Conclusion:**

To our knowledge, this is the first study to attempt a systematic review on this topic. The dearth of research in this field of enquiry is reflective of unique challenges associated with assessments of humiliation and shame in various clinical settings amongst adolescent and young adult populations. Nonetheless, given the importance and relevance of the psychological imprint of humiliation in youth morbidity and mortality in the field of mental health, it is timely to attempt such a systematic review. In light of the associated role of humiliation and shame in self-harm and suicidality among young people, we recommend that these processes need to be explored further via prospective studies and assessed as part of a comprehensive bio-psycho-social assessment when focusing on life stressors for adolescent and young adults presenting with suicidality to emergency departments and mental health services.

## Introduction

Suicidal behaviour is a leading cause of death and disability worldwide [1, 2]. While the interpretation of what suicidal behaviour and suicidality mean or represent varies generally, these terms refer to any thoughts or actions related to suicide, regardless of whether they involve planning or result in non-fatal attempts or death [1]. There is no universal consensus regarding the terminology related to suicidality or suicidal behaviour[2]. The term ’self-harm’ is used to refer to both intentional self-injury without the intent to die (i.e., non-suicidal self-injury behaviours such as superficial skin cutting) and concomitantly used to denote all intentional self-injurious behaviours regardless of intent to die [1]. Thus, self-harm is defined as a non-fatal act in which a person harms themselves, and the intent to die is either absent or not accessible to observation [2]. Non-suicidal self-injury (NSSI) is the term proposed in the DSM-5 and reflects an intentional behaviour that is not socially accepted (to distinguish it from tattoos and piercings) and leads to the destruction or injury of body tissue without major physical harm [3]. NSSI is the direct, deliberate destruction of one’s own body tissue in the absence of suicidal intent [4]. The presence of intent to die is central to the definition of a ’suicide attempt,’ a behaviour in which a person harms themselves with the intention to die but survives [2]. Suicidal ideation, often called suicidal thoughts or ideas, is a broad term used to describe a range of contemplations, wishes, and preoccupations with death and suicide [5].

Suicidal behaviours and death by suicide remain a major public health concern for adolescents and young adults across the world [6, 7]. Suicide is the second leading cause of death among young people worldwide, with several countries reporting increases in self-harm among this cohort in recent years [8, 9]. Globally, suicide is the leading cause of death for female adolescents and the third most common for male adolescents after road traffic accidents and violence [10]. Suicidal behaviour becomes increasingly common after puberty. This is a trend likely attributable to new-onset mood disorders and substance abuse [11], school/family problems, conflictual peer relations, adverse early childhood experiences [11], and evolving personality factors including neuroticism and impulsivity [12]. Cognitive immaturity, lack of judgment and low impulse control play an important role in this increased risk of suicide [11], with research demonstrating an association between adolescent neurobiological changes and increased risk-taking behaviours [13]. Life stress is a critical factor in all major theories of suicide [14, 15]. Life stressors, including acute life events, chronic difficulties, discrimination [16, 17] and trauma are associated with both suicidal ideation and attempts in adolescence and adulthood [9, 11]. Evidence for an association between negative life events and suicidality is consistent [9, 13]. Many adverse experiences resulting from relationships, peer conflict, victimisation, and isolation are associated with suicidal behaviours [9, 18–21], with longitudinal research revealing that key social contexts in early adolescence, (involving familial and peer relationships), have far-reaching influences on levels of suicidal behaviours in later life [19].

Self-harm has been reported among the five leading causes of mortality for young people in Europe [22], with self-harm documented as the third leading cause of Disability-Adjusted Life Years (DALYs) among people aged 10–24 years worldwide in 2019 [22] Self-harm represents an ongoing major public health problem for adolescents, with high rates of self-injurious behaviour noted in teenage years in particular [6]. Hospital-presenting self-harm data over a 10-year period clearly indicates a peak rate of self-harm among adolescents (15 to 19-year-old females; 564 per 100,000) and young adults (20 to 24-year-old males; 448 per 100,000) [23]. During this 10-year period, rates of self-harm increased by 22%, with increases most pronounced for females and those aged 10 to 14 years [23]. In addition, there is an increased prevalence of self-harm repetition among these age groups in recent years, with some interesting age and gender variations [24]. Whilst self-harm repetition rate is high among adolescent females (15 to 19 years) in comparison to young adults; this repetition rate is also high among young adult males [24].

A review of 56 community-based surveys on self-harm in adolescents, covering the period from 1990 to 2015, indicated that the average lifetime prevalence of self-harm in this age group increased significantly over this 25-year period [25]. The vulnerability of adolescents to developing suicidality arises due to elevated levels of impulsivity and emotional reactivity resulting from brain developmental processes [26]. Consequently, adolescents may have a weaker ability to initiate positive adaptive responses to negative emotions, and are more susceptible to adopting self-harm as a coping mechanism, particularly when subjected to external stressors. A case in point is the implication of Coronovirus: Evidently, there was an increase in self-harm documented among adolescents during the COVID-19 pandemic [27], as reported by a recent systematic review which demonstrated that the pooled prevalence of self-harm was 22.9% in adolescents compared to 11.7% in other age groups [27].

The integrated motivational-volitional (IMV) model [28] is a widely accepted theory that explains suicidal behaviour as a process comprised of three phases, each involving different mediating factors. Although it mainly focuses on adults, this model is relevant to this systematic review. In the pre-motivational phase, biological, genetic, and cognitive factors make individuals vulnerable to suicidal behaviour [28]. For instance, decreased serotonergic neurotransmission and socially prescribed perfectionism are factors that pre-dispose individuals to self-harm or suicidal thoughts. The feeling of defeat intensifies when an interpersonal crisis occurs, and higher levels of perfectionism increase sensitivity to emotional pain. In adolescence, vulnerabilities in the pre-motivational phase include developing cognitions and maturing emotional processes, which impact on a young person’s interpretation of, and adaptive response to stressors. In the motivational phase, negative feelings such as defeat and humiliation influence the development of suicidal ideas and plans. A sense of entrapment develops when individuals feel that there is no escape from humiliation or defeat, which is a proximal predictor of suicidal ideation. In adolescence, heightened sensitivity to the negative sequelae of peer rejection or lack of social acceptance may contribute to the process of entrapment. While protective motivational moderators like social support and belongingness allow trapped individuals to see positive alternatives, feelings of burdensomeness and poor social support increase the risk of entrapment leading to suicidal ideation. In adolescence, if the valued source of social support from peers becomes diminished through rejection, (perceived or otherwise), a vital subjective buffer in this regard is potentially lost. Finally, in the volitional phase, precipitating factors such as access to means or impulsivity lead to a suicide attempt [28]. In adolescence, impulsivity plays an important role in risk-taking [29] and in suicidality in vulnerable young people.

Another important theoretical model in understanding the aetiology of suicid is the Interpersonal Theory proposed by Joiner [30]. This theory was further expanded upon by Van Orden and colleagues [31]. The Interpersonal Theory of suicide (IPTS) considers three variables: thwarted belongingness, perceived burdensomeness, and acquired capability, as well as two phases: suicide desire and suicide attempt. If an individual experiences thwarted belongingness or perceived burdensomeness separately, they may present with suicidal desire. However, to progress from suicidal ideation to a suicide attempt, a third variable, namely; acquired capacity, comes into consideration. Acquired capacity refers to the destruction of the survival instinct [30, 31]. A recent systematic review examined this association and concluded that the interaction between thwarted belongingness and perceived burdensomeness was significantly associated with suicidal ideation. Furthermore, the interaction between thwarted belongingness, perceived burdensomeness, and capability for suicide was significantly related to a greater number of prior suicide attempts [32].

Joiners theory has also proven useful in predicting suicide risk in the adolescent population [33]. While IPTS does not detail the specific role of humiliation or shame in relation to suicidality, it is still relevant to mention, as interpersonal stressors are central to this theory. During adolescence, socio-emotional processes that normatively occur during this period of development are implicated in a risk of progression towards suicidal thoughts and behaviours [34]. The period of adolescence is typified by changes in social-affective processing, with heightened sensitivity to perceived peer evaluation, acceptance and rejection, and an increased need for peer connection/affiliation and belongingness occurring in the context of evolving autonomy [34–37]. Given the challenges that this poses to the developing adolescent in crisis; humiliation and shame may play even more of a distilled role during periods of interpersonal stress in activating a suicidal process than it might in later adulthood. For instance, the experience of humiliation or shame may deepen the perception of social alienation or thwarted belongingness among adolescent peers, intensify the experience of perceived burdensomeness, and activate capability for self-harm. Adolescent-specific research conducted through the lens of IPTS indicates an interaction between processes of thwarted belongingness and perceived burdensomeness in predicting suicidal ideation, while acquired capability for self-harm was demonstrated to predict suicide attempt, albeit independently of suicidal ideation [38].

The role of humiliation/negative appraisal leading to a sense of inescapable entrapment in vulnerable individuals is a core feature of progression towards suicidality in the IMV model. We postulate that humiliation and shame may also play a role in intensifying thwarted belonging and perceived burdensomeness, leading to maladaptive coping which affects the two phases of acquired capability of suicidal desire and attempt in adolescence. What this means for adolescents and young adults is very relevant. In the context of negative life events, humiliation is the single most experienced life stress among adolescents followed by interpersonal loss [9], and thus warrants further attention. The process of ‘humiliation’ refers to two different forms of experience. Firstly; the act of humiliating or being humiliated, and secondly; the state or feeling of being humiliated. Essentially, humiliation can be considered as an external event or an internal state.

Humiliation is associated with many mental health conditions. Persistent fear of being humiliated or scrutinised by others is common in social anxiety disorder among adolescents [39], while the experience of severe public humiliation can lead to major depressive illness [40, 41], hopelessness, and helplessness [42], and is associated with suicidal ideation or acts [43].

Shame can be understood as a cognitive affective construct, comprised of negative judgements of the self [44], which are global, undesirable, and characterised by an evaluation of the self as inherently weak, inadequate, or *“bad”* [45, 46]. Shame is a subjective emotional response to negative events such as the making of mistakes, being wrong, and experiences of maltreatment [47, 48]. Shame plays a central component in psychosocial functioning in its role as a trans-diagnostic emotion associated with many mental health conditions [49].

Although shame and humiliation are often used interchangeably in literature [50], there are similarities and differences between these two constructs. Both shame and humiliation are categorized as "self-conscious emotions" [51], which require an individual to interpret an event as shameful or humiliating. However, humiliation involves more emphasis on an interaction where someone is degraded or forced into a lower position by someone who is more powerful at that moment [52]. In contrast, shame can be viewed as more of a general feeling of unworthiness or guilt that arises from a violation of one’s own moral or social standards. Therefore, while both emotions involve self-evaluation and consciousness of the self, humiliation is more specifically linked to the experience of being degraded or debased by someone else [50]. Klein [52], in clarifying the distinction between shame and humiliation avers that: *"Shame is what one feels when one has failed to live up to one’s ideals for what constitutes suitable behaviour in one’s eyes as well as the eyes of others*. *Humiliation is what one feels when one is ridiculed*, *scorned*, *held in contempt*, *or otherwise disparaged for what one is rather than what one does”* [52]. See Table 1 below, which summarises the major differences between shame and humiliation.

**Table 1:** The major differences between shame and humiliation.

Shame	Humiliation
Internal attribution	External attribution
Self as bad or flawed	Other as bad
Internal sense of inferiority	Internal sense of inferiority not necessary
Heightened self-consciousness	Greater focus on the other
No obvious sense of injustice	Strong sense of injustice
No strong desire for revenge	Strong desire for revenge

*Adopted from Gilbert (1997)* [53]

Given the fact that humiliation and shame have different predispositions and aetiology [44, 45], it is important to distinguish between these concepts in order to design the most appropriate psychotherapeutic interventions. Studies have associated shame with self-injurious behaviour [54], suicidal ideation [55] and suicide attempts [56]. Various systematic reviews have explored the connection between shame and numerous mental health issues. For instance, reviews have shown a positive link between shame and psychosis, albeit with partial support [45]. In the context of anorexia and bulimia nervosa, individuals with these disorders typically experience higher levels of shame compared to controls. Moreover, shame is positively correlated with the severity of symptoms and the onset of eating disorder-related difficulties [46]. In relation to depressive symptomatology, it has been observed that external shame yields stronger associations than internal shame [47] (External shame is centred on the experience of oneself being perceived in a judgmental manner by others, whereas internal shame is conceptualized as self-directed negative evaluations and feelings about the self) [57]). Furthermore, research indicates a significant association between shame and substance use behaviour over shorter periods of time [58], although its predictive reliability diminishes over longer time-frames. Only one systematic review has studied the association between shame and self-harm in adults [59]. This indicated that individuals with a history of self-harm reported greater shame, and highlighted a correlation between shame and frequency of self-harm [59]. However, this latter work did not focus on young people, and centres on self-harm outcomes only.

Therefore, given the paucity of systematic review evidence on the associations of shame and humiliation with suicidal outcomes among young people, the current review aimed to examine the prevalence of, and association between humiliation/shame and suicidal behaviours and/or suicide among adolescents and young adults.

## Methods

### Protocol registration

This systematic review followed the Preferred Reporting Items for Systematic Reviews and Meta-Analysis guidelines (PRISMA) [60] (See S1 Checklist). The protocol was registered with the International Prospective Register of Systematic Reviews (PROSPERO) [CRD42022289843], and subsequently published [61]. Departures from protocol include an exclusion of meta-analysis. The decision to exclude a meta-analysis was based on the following premises: a) lack of comparison group and high-risk of bias among most of the humiliation studies, b) issues with the objective measurement of exposure (only in humiliation studies) and outcome, and c) heterogeneity of instruments used to measure shame, with dimensions/subscales of these instruments hugely variable and essentially incomparable. Thus, we conducted a narrative synthesis, which we evaluated as more meaningful in the given context.

### Eligibility criteria

All original empirical studies published in English were considered for this review. Peer reviewed articles and grey literature were included. Specifically, the following eligibility criteria were applied:

### Study design

Quantitative research studies comprising cross-sectional, prospective or longitudinal, and case control studies were included. Mixed method studies were considered if quantitative measurements were included in the study variables. Experimental/quasi-experimental studies were added if sufficient baseline data were available. Qualitative studies, case reports, and case series were excluded.

### Participants

Adolescent or young adults (13–24 years of age).

### Exposure and outcome

Studies reporting the prevalence or association of humiliation or shame with self-harm, suicidal ideation, suicide attempts, and suicide (individuals who died by suicide) were included. Humiliation and/or shame measured by standard instruments, or self-reported questionnaire, or measured by single item/questions was included.

### Setting

No restriction by type of setting.

### Date of publications

While there were no restrictions on the date of publications, the last search was run on the 22^nd^ of March 2023.

### Information sources

Electronic databases including MEDLINE, Web of Science Core Collection, CINAHL, PsycINFO, and Embase were systematically searched to identify potential studies. In MEDLINE, we conducted ’all field’ searches, while in all other electronic databases, we performed ’title/abstract’ searches. Google Scholar (as a secondary source) was searched (first 200 articles) to identify if any potential studies had been left out. The combination of Embase, MEDLINE, Web of Science Core Collection, and Google Scholar performed best, achieving an overall recall of 98.3 and 100% recall in 72% of systematic reviews [62]. The thesis and dissertation database ProQuest was searched to identify relevant studies in the grey literature. Additionally, the reference list of the retrieved articles and/or previous systematic reviews in this area were also scanned to identify further potential studies. The literature search was originally conducted from 20^th^ September 2021 to 29^th^ April 2022. However, the search was updated on 22^nd^ March 2023.

### Search strategy

Based on our initial literature review of electronic databases, we identified a set of relevant search keywords that pertain to our review’s population, exposures, and outcomes. Specifically for the MEDLINE search, we matched these search terms to corresponding MeSH terms found in the MeSH library. We scrutinized the definitions of the selected MeSH terms in detail to ensure they accurately represented the intended concepts. In the MEDLINE search, we employed a combination of both MeSH terms and key terms. This literature search was conducted using a mix of these terms, including at least one term from each category (see the details below). Boolean operators such as ‘AND’, ‘OR’, ‘NOT’ were used to maximise the penetration of terms searched, and appropriate “wild cards” were employed to account for plurals, variations in databases, and spelling.

#### Category 1

*Population*. Adolescent (MESH), young adult (MESH), teen, teenage.

#### Category 2

*Exposure*. Humiliation, degradation, shame (MESH) or embarrassment (MESH), harassment, victimisation, abasement.

#### Category 3

*Outcome*. Self-injurious behaviour (MESH), suicide (MESH), suicide attempted (MESH), suicide completed (MESH), self-harm, intentional self-injury, deliberate self-harm, overdose, deliberate self-poisoning, non-suicidal self-injury, self-mutilation, suicidal thought, suicidal ideation, suicidal intent.

Details of the search strategy is included as a S1 File.

### Data management

The literature search results (including citations, abstracts and full text) were uploaded to Rayyan, an open source for the management of records for systematic reviews, where duplicates were removed.

### Study selection process

Two authors (AS & AMcL) independently screened the titles and abstracts yielded by the search against the inclusion criteria through Rayyan. We obtained full reports for all titles that appeared to meet the inclusion criteria, or where there was any uncertainty. Two of the review authors (AS & AMcL) then screened the full text reports and decided whether these met the inclusion criteria. Disagreement was resolved through discussion. Reasons for excluding studies were recorded through Rayyan.

While the majority of included studies were agreed upon by the two reviewers, further discussions were necessary for two studies. For instance, the study by Rolland et al. [63] measured both humiliation and suicidal thoughts, providing an association between humiliation and depressive symptoms. However, it did not offer a specific connection between humiliation and suicidal thoughts, nor did it present the prevalence of suicidal thoughts among those experiencing humiliation. After extensive deliberation, we decided to exclude this study. Similarly, the mixed methods study by Brown et al. [64] included quantitative measurements for both shame and self-injury, yet the sample size was small (n = 6), and the emphasis was on qualitative exploration rather than reporting a quantitative association. Thus, this study was also subject to rigorous discussion between the reviewers before a decision was reached.

### Data collection process

The relevant study details were populated onto a pre-prepared data extraction sheet on Microsoft Word. The self-prepared data extraction sheet included: the author, year of publication, country of study, study setting, population and sample, study design, outcome variables or measures, and main findings. Additionally, data relevant to methodological quality appraisal were extracted from all of the included studies. Data were extracted by two independent review authors (AS & AMcL).

### Risk of bias assessment

The studies were assessed for methodological quality using the Joanna Briggs Institute (JBI) critical appraisal tools for analytical cross-sectional (eight-item) [65], cohort studies (eleven-item) [66] or case control studies (ten-item) [67]. The items included assessment on sampling, study setting, measurement of exposure, condition and outcome, identification and management of confounding factors, appropriateness of the statistical methods, and three additional items for cohort studies (i.e. duration of follow-up, dropouts, and strategies to address incomplete follow-ups) and two additional items for case control studies (appropriate match of the case and control subjects, and the criteria used for identification of cases and controls). Each item in the JBI appraisal tools is answered as Yes, No, Unclear or Not applicable. Two review authors (AS & AMcL) independently applied the tool to each included study, and recorded supporting information and justifications for judgements of risk of bias for each domain. Any discrepancies in judgements of risk of bias or justifications for judgements were resolved by discussion to reach consensus between the two review authors, with a third review author acting as an arbiter if necessary.

### Data synthesis

We conducted a narrative synthesis. The summary of the studies and risk of bias assessment results were presented in tabular form in chronological order, starting from most recent. A descriptive summary of the findings including study country, setting, population and sample, design, exposure and outcome measures, and main findings were provided. The studies were narratively compared with reference to risk of bias, sample size, quality of measurement and exposure and outcomes.

## Results

### Study screening and selection

**Fig 1:**
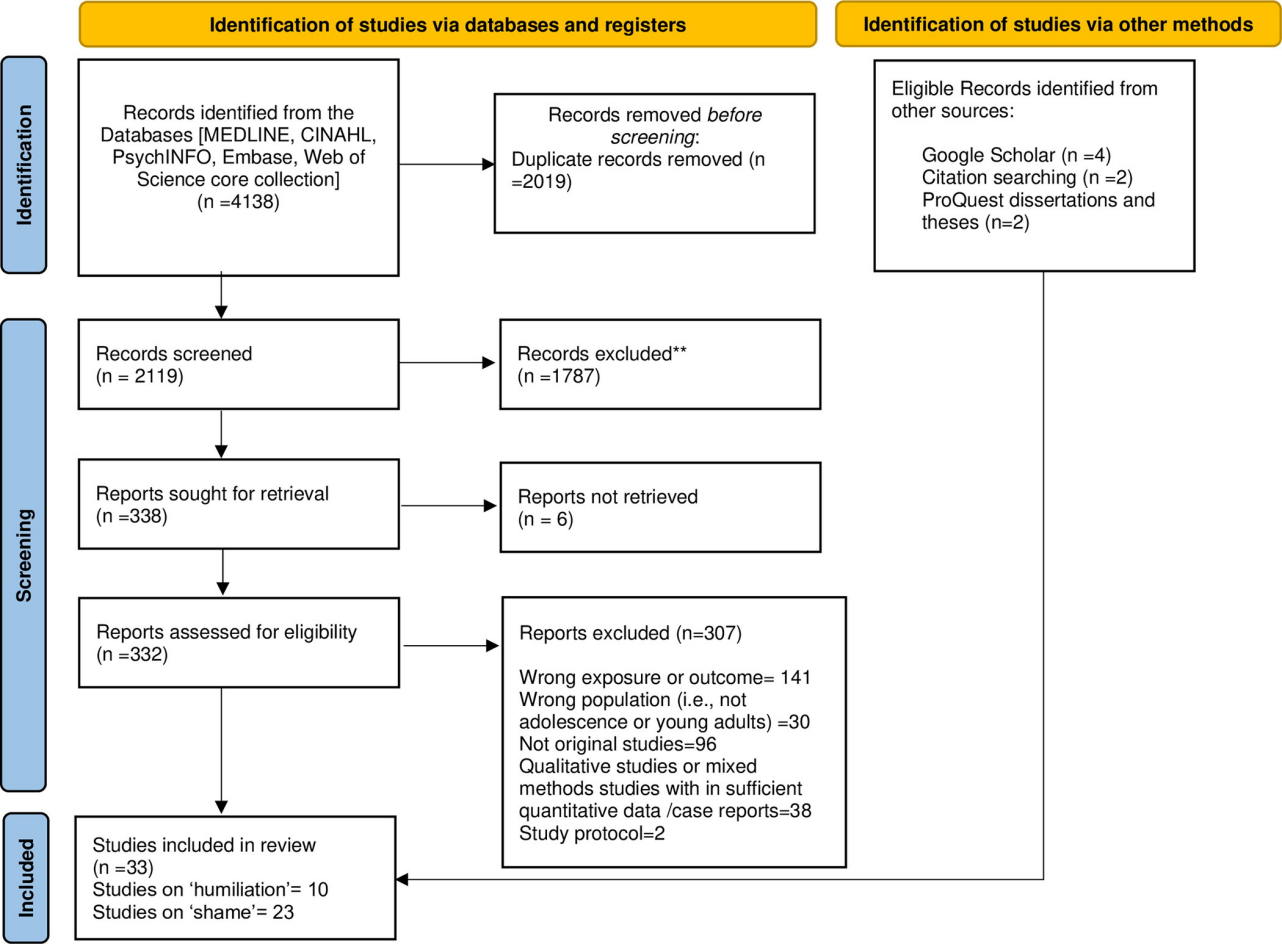
Study screening and selection process.

The initial stage of the article screening process involved identifying a total of 4,138 records from various databases, including MEDLINE, CINAHL, PsychINFO, Embase, and the Web of Science core collection. Among these records, 2,019 were removed due to duplication, resulting in 2,119 records remaining for further screening. During the screening phase, a rigorous evaluation was conducted, leading to the exclusion of 1,787 records. Reports were then actively sought for retrieval, with 338 reports fitting the criteria. However, 6 reports were not successfully retrieved. A detailed eligibility assessment was conducted on 332 reports. This assessment resulted in the exclusion of 307 reports for various reasons such as incorrect exposure or outcome criteria (141 records), instances where the studied population did not align with the focus on adolescence or young adults (30 records), and instances where the studies were not original research (96 records), including qualitative studies, case reports, and study protocols (38 and 2 records, respectively). In addition to the main databases, other sources were also explored. This included 4 eligible records found in Google Scholar, 2 records through citation searching, and 2 records from ProQuest dissertations and theses. Ultimately, the review included a total of 33 studies that met the eligibility criteria. Among these, 10 studies focused on ’humiliation,’ while 23 studies centred on ’shame’ (See Fig 1).

### Methodological quality assessment

Four studies examining humiliation [68–71] scored 50% or less in the current methodological quality assessment, indicating high-risk of bias in the data reviewed. Notably, poor scores were attributed to JBI Checklist items such as valid exposure measures, identification of confounding factors, or strategies to deal with the confounding factors (see Table 2). The use of non-validated instruments or a single dichotomous item for measuring the exposure (i.e., humiliation) was the most conspicuous shortcoming. As Stewart et. al.’s (2019) study was a case control design, we therefore also used the JBI case control study checklist, which includes 10 items [72]. This study scored 9, indicating a low risk of bias (not included in the table).

**Table 2:** Critical evaluations of the cross sectional studies on humiliation and suicidal behaviours.

Study	Were the criteria for inclusion in the sample clearly defined?	Were the study subjects and the setting described in detail?	Exposure measured in a valid and reliable way?	Objective, standard criteria used for measurement of the condition?	Confounding factors identified?	Strategies to deal with confounding factors stated?	Outcomes measured in a valid and reliable way?	Appropriate statistical analysis used?	JBI score
Ellison et al (2022) [87]	Y	Y	Y	Y	N	N	Y	Y	6
Rasheduzzaman et al (2022) [71]	Y	Y	N	Y	N	N	N	Y	4
Crawford et al, (2019) [86]	Y	Y	Y	Y	Y	Y	Y	Y	8
Sahoo, Biswas& Agarwal (2018) [69]	Y	Y	N	N	N	N	Y	N	3
Sahoo, Biswas& Agarwal (2016) [68]	Y	Y	N	N	N	N	Y	N	3
Christoffersen and Depanfilis, (2010) [86]	Y	Y	N	Y	Y	Y	Y	Y	7
Toros et al, (2004) [88]	Y	Y	N	Y	Y	Y	N	Y	6
Valentiner, Gutierrez, Blacker (2002) [89]	Y	Y	Y	Y	Y	Y	Y	Y	8
Apter et al, (1993) [70]	Y	Y	N	N	N	N	Y	Y	4
**Subdomain scores**	9	9	4	8	5	5	8	8	---

Of twenty cross-sectional studies/data on shame and suicidality, sixteen studies have scored 6 or above (75% or above) indicating a very good methodological quality or low risk of bias. One major issue across the studies were the use of non-validated instruments utilised in measuring the outcomes (i.e., suicidal behaviours) (see Table 3). Three longitudinal studies on shame and suicidal behaviours [81–83] were assessed by the 11-item JBI critical appraisal tool for cohort studies [84]. Among these, one study [83] scored 9, indicating high-methodological quality (although sample size was reduced), with two other studies [81, 82] scoring 7, albeit this score was attained in the absence of a comparison group (not included in the table).

**Table 3:** Critical evaluations of the cross sectional studies on shame and suicidal behaviours.

Study	Were the criteria for inclusion in the sample clearly defined?	Were the study subjects and the setting described in detail?	Exposure measured in a valid and reliable way?	Objective, standard criteria used for measurement of the condition?	Confounding factors identified?	Strategies to deal with confounding factors stated?	Outcomes measured in a valid and reliable way?	Appropriate statistical analysis used?	JBI score
Grove et al (2023)* [99]	Y	N	Y	Y	N	N	Y	Y	5
Schneider et al, (2022) [55]	Y	Y	Y	Y	Y	Y	N	Y	7
Nicol, Mak, Murray & Kavanagh (2021) [77]	Y	Y	Y	Y	Y	Y	N	Y	7
Zhao et al (2020) [56]	Y	Y	Y	Y	Y	Y	N	Y	7
Sekowski et al (2020) [97]	Y	Y	Y	Y	Y	Y	Y	Y	8
Mahtani, Hasking, Melvin (2019) [100]	Y	N	Y	Y	Y	Y	Y	Y	7
Keng et al (2019) [98]	Y	Y	Y	Y	Y	Y	Y	Y	8
Ellenbogen et al (2018) [78]	Y	Y	N	N	Y	Y	N	Y	5
Alix et al (2017) [73]	Y	Y	Y	Y	Y	Y	N	Y	7
Garcia et al (2017) [92]	Y	Y	Y	Y	Y	Y	Y	Y	8
Xavier, Pinto Gouveia, & Cunha (2016)	Y	Y	Y	Y	Y	Y	Y	Y	8
Wong et al (2014) [93]	Y	Y	Y	Y	Y	Y	Y	Y	8
Schoenleber (2013) [94]	Y	Y	Y	Y	Unclear	Unclear	Y	Y	6
Unikel, Von Holle, Bulik, Ocampo (2012)	Y	Y	Y	N	N	N	N	Y	4
Nelson & Muehlenkamp (2012)	Y	Y	Y	Y	Y	N	N	Y	6
Flett, Goldstein, Hewitt & Wekerle, (2012) [75]	Y	Y	Y	Y	Y	N	N	Y	6
Kahumoku et al (2011) [76]	Y	Y	Y	Y	Y	Y	Unclear	Y	7
Mcleod (2003) [95]	Y	Y	Y	Y	Y	Y	Y	Y	8
Lester, 1998	N	Y	Y	Y	Y	Y	N	Y	6
Wright and Heppner, (1991) [96]	Y	Y	Unclear	Unclear	Unclear	Unclear	Y	Y	4
**Subdomain scores**	19	18	18	17	16	14	10	20	--

Note

* Grove et al.’s study was originally a pre-post assessment, and we included this study here as we utilized the baseline data only

### Characteristics of the included studies

A total of thirty-three studies reporting the prevalence or association between humiliation (n = 10) or shame (n = 23) and suicidality were included in this review. Study characteristics are summarised in Tables 4 & 5.

**Table 4:** Summary of studies on the prevalence and or association of humiliation and suicidal behaviour.

Author	Study setting and country	Population and sample	Study design	Measures/ variables	Prevalence of humiliation	Association between humiliation and suicidal behaviours
Ellison et al (2022) [87]	Yale Child Study Center in New Haven, Connecticut, US	N = 48 children with a diagnosis of ASD 8–17 year old *Mean age* = 13.77 SD = 2.84	Cross sectional Two groups: children with suicide intent (n = 9) and without suicide intent (n = 39)	Suicide intent = Columbia Suicide Severity Rating Scale [103] Humiliation = MASC‑C and MASC‑P scale [118]	**------**	MASC-P subscale score was significantly higher among children with suicide intent. However, thewas no significant group differences on MASC-C subscale score
Rasheduzzaman et alx (2022) [71]	Undergraduate university students, Bangladesh	N = 1844 70% males Mean age = 20.92 (SD)±1.72)	Cross sectional	Binary categorical items to measure past year stressful life events, and suicidal behaviours	Humiliating experience in the form of campus ragging-29.2%	Associated with SI (χ2 = 27.523, *p<*0.001) and SP (χ2 = 9.771, *p<*0.001). Not associated with SA.
Stewart et al, (2019) [14]	Recruited from psychiatric short-term residential care. USA	197 (144 female) adolescents aged 13 to 19 years old (M = 15.61, SD = 1.48). Groups: PC (n = 38) SI (n = 99) and SA (n = 60)	Case control	Stress and Adversity Inventory [119] which include a subscale for humiliation. The instrument differentiates acute life events (episodic) and chronic (persistent) difficulties	-------	No significant group differences. Humiliation (chronic difficulties) was significantly higher in SA (M 0.88, SD 0.88) and SI (M 0.75 SD 0.79), than PC (M 0.45, SD 0.55) in un-adjusted model.
Sahoo, Biswas& Agarwal (2018) [69] India	Hospital India	101 adolescent suicide attempters, referred from the medical ward and causality	Cross sectional	A semi structured interview schedule was used to record various risk factors for suicide attempt. Perceived humiliation measured with a single item. Suicide intent was measured by Pierce’s suicide intent scale	18% of adolescent reported humiliation experience before the suicide attempt.	----
Crawford et al, (2018) [86]	Outpatient specialty clinic for anxiety disorders. USA	87 Children and adolescents ages 6–17 (M = 11.1 years, SD = 3.06; 52.9% male) diagnosed with a principal anxiety disorder	Cross sectional	Suicidal Ideation Questionnaire-Junior. MASC, which included a subscale for humiliation/rejection	------	Humiliation subscale score was associated with child reported suicide intent in bivariate analysis (r = 425; p < .001.). They were not associated when adjusted for the confounders
Sahoo, Biswas& Agarwal (2016) [68]	Hospital India	32 adolescents	Cross sectional	A semi structured interview schedule was used to record various risk factors for suicide attempt. Suicide intent was measured by Pierce’s suicide intent scale Perceived humiliation measured with a single item	28.1% adolescences reported humiliation prior to the episode.	------
Christoffersen and Depanfilis, (2010) [90]	Based on the records obtained from child protection agencies. Denmark	1055 children at risk (children referred to child protection agencies). Age range from 0–22 years.	Cross sectional analysis of the data obtained from the child protection agencies	Self-prepared dichotomise items to measure psychological maltreatment which included humiliating speech to a child. Suicidal behaviour included attempted suicide and contemplated suicide	Prevalence of psychological maltreatment including humiliation among those with a history of suicidal behaviour was 67.1% (n = 76).	Significant association between caregiver psychological maltreatment and suicide attempts in children (OR = 4.2; p = .0001)
Toros et al, (2004) [88]	School based study, 6^th^-11^th^ Grades. Turkey	4143 students. Participants were divided in to two groups according to the experience (n = 80) and non-experience of suicide attempt	Cross sectional	A self-prepared structured questionnaire which collects details on various risk factors for suicide	------	Humiliation was one of the strongest risk factors for suicide attempt (OR = 3.85; P =. 0000)
Valentiner, Gutierrez, Blacker, (2002) [89]	Ethnically diverse high-school in a small Mid-west city. USA	138 High-school students. 14–18 years	Cross sectional	MASC [118]-Humiliation /Rejection subscale Suicide ideation questionnaire (SIQ) Self-harmful behaviour scale	Mean humiliation/rejection score was 2.2 (SD 0.8).	There was no correlation between humiliation/rejection subscale and suicidal intent or self-harm measures
Apter et al, (1993) [70]	Suicide occurred during compulsory military services. Israel	Forty-three consecutive Israeli male suicides, 18 to 21 years of age	Cross sectional. Also included retrospective information collected via post-mortem records	Post-mortem interviews with family and peers for understanding various stressor which have led to suicide	Service related stresses (28%) (stress predominantly resulted from humiliation or insult)	-------

ASD = Autism Spectrum Disorder; MASC = Multidimensional Anxiety Scale for Children; MASC‑C and MASC‑P = Multidimensional Anxiety Scale for Children, Child‑Report and Parent‑Report; PC = Psychiatric Control; SI = Suicide Ideation; SA = Suicide Attempt; SP = Suicidal Plan

**Table 5:** Summary of studies on shame and suicidal behaviour.

Author	Study setting and country	Population and sample	Study design	Measures of exposure	Measures of outcomes	Association between shame and suicidal behaviours
Grove et al (2023) [99]	Recruited from inpatient and outpatient units, community and undergraduate psychology pool, US	N = 72 young adults Mean age = 24.02 (SD 5.23) Female = 66% Two groups: Lifetime NSSI history group Non-NSSI group	Secondary analysis of a pre-test post-test study. Baseline data only included in this review	PANAS [120]	SITBI [121]	No significant correlation between NSSI history and shame reactivity (baseline)
Schneider et al, (2022)[55]	Four Cuban high schools and a psychiatric day hospital, Cuba	N = 844 adolescence Median age = 16 years Females = 64.3% Three groups: hospital attempters (HA), community attempters (CA), and community non-attempters (CN).	Cross sectional	ESS [122]	SIS [123], Suicide attempts, PANSI [124]	Significant differences between attempters and non-attempters in all three categories of shame including character shame, behavioural shame and bodily shame
Dyer, Goodman, & Hardy (2022) [81]	Data came from the Family Foundations of Youth Development Project, Utah, USA	N = 617 adolescence 11–15 years of age	Longitudinal study. Wave 1 at 2016 Wave 2 at 2018	ISS [125]	SI was assessed with a single binary categorical item	Wave 1 shame score was correlated with SI at W2 (r = .43), In the path analysis, shame at wave 1 predicted suicidal ideation at wave2. (B(SE) .24(.10), p .05; β = .19)
Nicol, Mak, Murray & Kavanagh (2021) [77]	Secondary and university students, Australia	N = 403 16–25 years Female = 66.5% Mean age of Secondary students = 16.64 (SD = 0.64) Mean age of university students = 20.38 (SD = 2.29)	Cross sectional	YSQ‐S3, which include a subscale for measuring shame/defectiveness	NSSI was assessed using a single dichotomous screening question	In bivariate analysis, shame was correlated with NSSI (r = 0.34) In multiple logistic regression model, shame predicted NSSI history (OR = 1.64)
Alix et al (2020) [82]	Four CSA intervention centres in the province of Quebec, Canada	N = 100 Sexually abused adolescent girls, 14–18 years of age	Longitudinal study Baseline (T1) and 6-month follow-up (T2)	ASSQ [126]	Single Likert type item to measure SI	Shame (M 3.96 SD2.53) atT1 correlated with SI at T2 (r = .474**) However, shame at T1 did not predict SI at T2 in the path analysis
Sekowski et al (2020) [97]	Adolescent unit of a private psychiatric hospital, US	N = 112 inpatient adolescents Aged 12–17 years 73 girls and 39 boys	Cross sectional	PFQ-2 [127]	C-SSRS) [103]	Generalized Shame (M 21.38 SD 8.16) correlated with intensity (r_s_ = .508) and severity (r_s_ = .412) of suicidal ideation. In the path analysis, generalised shame increased the intensity and severity of suicidal ideation via depressive symptoms
Zhao et al (2020) [56]	Undergraduate students in a university, China	N = 2320 Young adults Gender almost equally distributed	Cross sectional	MSRI–21 [92]	SI was assessed with a single binary categorical item	Shame M 45.49; SD17.70 positively correlated with SI. r = 0.185 In the multivariate analysis, shame predicted suicidal ideation (OR = 1.02, p < 0.001)
Mahtani, Hasking, Melvin (2019) [100]	Australia	N = 573 emerging adults. Mean age = 20.7 Female = 69.1% NSSI history: n = 220	Cross sectional	TOSCA-3 [128] CoSS-5 [129]	ISAS [130]	Shame proneness was correlated with NSSI (r = 0.39).Shame proneness (OR = 4.03), Shame coping-attack self (OR = 3.71) and shame coping-withdraw (OR = 3.50) predicted NSSI in past 12 months.
Keng et al (2019) [98]	Probation and community rehabilitation service, Singapore	N = 100 adolescent offenders Age = 13–20 years Mean age 17.5 years Males = 85%	Cross sectional	Adolescent Shame Measure [131]	NSSI was assessed using FASM 23 [132].	Shame score (M 24.31; SD 10.75) was correlated with past year NSSI. Shame significantly mediated the association between high betrayal trauma and NSSI
Ellenbogen et al (2018) [78]	Data derived from Maltreatment and Adolescent Pathways (MAP) project, Canada	N = 287 Mean age = 15.9 Female = 56.1%	Cross sectional data of a longitudinal study	Physical abuse (PA) and sexual abuse (SA) shame was measured using a subscale of SQ [126].	Suicide ideation was measured suing a 3-itme self-prepared questionnaire	PA -shame was significantly associated with suicidal ideation in the multivariate model (r2 = 0.05).
Alix et al (2017) [73]	Four CSA intervention centres in the province of Quebec, Canada	N = 147 sexually abused adolescent girls Age = 14–18 years	Cross sectional data of a longitudinal study	ASSQ [126]	Single Likert type item to measure SI	Shame was correlated with suicidal ideation (r = .398). In path analysis, shame directly predicted suicidal ideation. Shame also found to partially mediate the relationship between self-blame and suicidal ideation.
Garcia et al (2017) [92]	Psychology undergraduate students at a public university in the Southwestern, US	N = 540 Females = 69.8% Mean age 19.48 (3.32)	Cross sectional-scale development study	MSRI-21 [92]	FASM-23 [132] SBQ-R) [133]	Two subscales of MSRI-21 were strongly associated with FASM and SBQ scores.
Xavier, Pinto Gouveia, & Cunha (2016) [91]	Middle and secondary schools in the district of Coimbra, Portugal	N = 782 Adolescents with 12–18 years-old M = 14.89, SD = 1.76)	Cross sectional	External shame measured by OAS2 [134]	NSSI measured by RTSHIA [135]	External shame was correlated with NSSI (r = .39). Path analysis revealed a significant indirect effect of external shame on NSSI
Duggan, Heath, Hu (2015) [83]	15 high schools in Montreal, Quebec, Canada	N = 120 Female = 56% Age = 11–13 years (M = 12.34, SD = .48).	Longitudinal study. 12-months follow-up. Three groups NSSI Maintain, NSSI Stop, and Comparison group	OBCS- -Youth	A single item to screen the presence and absence of NSSI	At T1, the NSSI Maintain (M = 3.75; SD = 1.14) and NSSI Stop group (M = 3.47; SD = 1.07) reported significantly more body shame when compared to the comparison group (M = 2.68; SD = 0.87). No significant group differences on shame score at T12
Wong et al (2014) [93]	Undergraduate psychology subject pool in a large West Coast university, US	N = 476 Female = 65.8% Mean age = 20.02 (1.76)	Cross sectional scale development study	ISI [93]. Two subscales to measure external shame (ISI-E) and family shame (ISI-F)	Suicide Ideation Scale [136] SSS [137]	Bivariate correlation between suicidal ideation and ISI-E (r = .46), and ISE-F (r = .52). SSS score was also correlated with suicidal ideation (r = .54). In path analysis, ISI-E and ISI-F directly associated with suicidal ideation.
Schoenleber (2013) [94]	Undergraduate psychology students and women currently living in the Champaign- Urbana community, US	N = 115 Self-injury group = 26 Non-self-injurious group = 89 Mean age = 18.9 (SD = 0.8) years Females only	Cross sectional	TOSCA-3 [128]. ShARQ [138]	ISAS [130]	Shame proneness and SI (rho = .24) Shame aversion and SI (rho = .37) In the binary logistic regression, shame proneness (OR = 2.12) and shame aversion (OR = 1.44) predicted the presence of SI
Unikel, Von Holle, Bulik, Ocampo (2012)	High school students in central Mexico	N = 2357 (females) Mean age = 16.27 (0.03)	Cross sectional	Body shame measured by TOSCA-A.	Suicide intent measured with a questionnaire developed by Gonza´lez-Forteza et al., 2002 [139].	Shame was associated with suicide intent (OR = 1.05) in un-adjusted model
Nelson & Muehlenkamp (2012) [74]	Undergraduate university students, USA	N = 341 Female = 82.4% Mean age = 20.2 (SD = 1.98)	Cross sectional	OBCS [140]	DSHI [141]	Body shame correlated with self-injury (rho = .20) Individual with a history of self-harm reported significantly higher body shame (Mean = 4.05; SD .12) than those without the self-harm history (Mean = 3.46;SD = .07)
Flett, Goldstein, Hewitt & Wekerle, (2012) [75]	University students, Canada	N = 319 Mean age 18.89 (SD 2.30) years Female = 65%	Cross sectional	ESS [142], which measure characterological, behavioural and bodily shame	Self-harm Inventory (SHI) [143]	Characterological shame (r = .28), bodily shame (r = .28) and total shame score (r = .25) were correlated with SHI score of women participants
Kahumoku et al (2011) [76]	School settings, Switzerland and Georgia, US.	Samples from Switzerland N = 3803 females mean age = 17.81 16–20 years Samples from Georgia N = 2657 females Mean age = 16.44 16–20 years	Cross sectional	OBCS [140], which include a subscale for measuring body shame	Suicidal ideation was assessed by a 5-item questionnaire	In path analysis, body shame was significantly associated with suicidal ideation in Swiss (b = 1.90; (SE .11) β = .32; < .001) and Georgian samples (b = 1.55; (SE .16) β = .30; < .001) samples. Swiss youth reported significantly more body shame than Georgian adolescents.
Mcleod (2003) [95]	Undergraduate psychology students, Canada	N = 232 Mean age = 19.2 (SD 1.0) years Female = 73.7%	Cross sectional scale development study	TOSCA [144] DSQ [95] (relationship shame (RS), appearance shame (AS) and performance shame (PS)) PFQ-2 [145]	Suicide ideation (SI) and behaviour questionnaire [146]	DSQ-PS correlated with SI (r = .32) and suicide attempts (r = .20) DSQ-AS correlated with SI (r = .39) and suicide attempts (r = .26). DSQ- RS correlated with SI (r = .34) and suicide attempt (r = .26). In multiple regression, DSQ-AS and PFQ-2 Shame proneness predicted SI
Lester, 1998 [80]	Undergraduate students, US	N = 116 Mean age = 21.9 (SD = 4.6) years Female = 67.2	Cross sectional	DCQ [147]	Beck Depression Inventory, which contain one item measuring current suicidality	Shame scores were associated significantly with current suicidality (rs = .22) The association between shame and current suicidality was present for the males but not for the females.
Wright and Heppner, (1991) [96]	Undergraduate psychology students (children of alcoholics) at a large midwestern university, US	N = 80 Female = 50% Mean age = 18 Range = 18–21 years	Cross sectional	SPQ by Shreve & Patton (1987) (Reference not available)	SSI [123]	SPQ scores were not associated with SSI

*ASSQ = Abuse Specific Shame Questionnaire; CoSS = Compass of Shame Scale; CSA = Child sexual abuse;C-SSRS = Columbia-Suicide Severity Rating Scale; DCQ = Dimensions of Conscience Questionnaire; DSQ = Domains of Shame Questionnaire; ESS = Experience of Shame Scale; DSHI = Deliberate Self-Harm Inventory; ESS = Experiential Shame Scale; FASM = Functional Assessment of Self-Mutilation; ISAS = Inventory of Statements about Self-Injury; ISI = Interpersonal Shame Inventory; ISS = Internalized Shame Scale; MSRI = Multidimensional Shame Response Inventory; NSSI = Non-suicidal self-injury;OBCS = Objectified Body Consciousness Scale; OAS = Other as Shamer Scale;* PANAS = Positive and Negative Affect Schedule *PANSI = Positive and Negative Suicide Ideation Inventory; PFQ = Personal feeling questionnaire; PFQ = Personal Feelings Questionnaire; RTSHIA = Risk-Taking and Self-Harm Inventory for Adolescents; SBQ-R = Suicidal Behaviors Questionnaire Revised; ShARQ = Shame-Aversive Reactions Questionnaire;SI = Suicidal Ideation; SIS = Suicide Intent Scale; SITBI =* Self‐Injurious Thoughts and Behaviors Inventory; *SPQ = Shame-Proneness Questionnaire; SQ = Shame Questionnaire; SSI = Scale for Suicidal Ideation; SSS = State shame scale; TOSCA = Test of Self-Conscious Affect; TOSCA-A = Test of Self-Conscious Affect-Adolescent; YSQ-S = Young Schema Questionnaire Short form;*

### Study country, setting and population

The majority of the studies on humiliation were from the USA (n = 4) and India (n = 2). Five studies were conducted in hospital settings and included adolescents with a history of self-harm [68, 69, 85] and/or suicidal ideation [85–87]. Samples were identified from medical wards [68, 69], short-term residential psychiatric care [85], an outpatient speciality clinic for anxiety disorders [86] or a child psychiatric unit [87], and included children and adolescent with autistic spectrum disorder [87]. Three studies were conducted in community settings, which included school children [88, 89] and university undergraduate students [71]. One study was based on the records obtained from child protection agencies [90], while another study included post-mortem interviews with family members [70]. Most of these studies primarily focussed on adolescents [68, 69, 85, 89], or included adolescents and children [86, 87, 88, 90] or young adults [70, 71].

More than 40% of the studies on shame and suicidality(n = 10) were conducted in the United States, followed by Canada with over one-fourth (n = 6) and Australia with two studies. More than half of these studies were conducted in educational settings, with samples consisting of either secondary school students [55, 76, 77, 79, 83, 91] or undergraduate university students [56, 74, 75, 77, 80, 92–96]. Two studies collected samples from psychiatric hospitals [55, 97] while three studies collected samples from intervention centres for child sexual abuse [73, 82] or maltreatment [78]. One study collected samples from probation and community rehabilitation services for children [98], and another recruited participants from a range of settings, including inpatient and outpatient units, community centres, and undergraduate psychology pools [99]. The studies primarily focused on adolescent populations [55, 73, 75, 76, 78, 79, 81, 82, 91, 97, 98] or adolescents and children [83] or adolescents and young adults [56, 74, 77, 92–95, 100] or predominantly with young adults [80, 96, 99].

### Sample size

For studies on humiliation, the sample size varied greatly, ranging from n = 32 [68] to n = 4143 [88]. Although there were three large sample studies with samples of n = 1055 [90], n = 1844 [71] n = 4143 [88], six other studies had sample sizes of n = 200 or less. The sample size of the five hospital-based studies ranged from n = 32 [68] to n = 197 [85], while for community-based studies, it ranged from n = 138 [89] to n = 4143 [88]. In one study only males were included [70], while in three studies, two-thirds of the samples were comprised of boys/males [71, 68, 90].

For studies on shame, sample sizes ranged from n = 80 [96] to n = 3803 [76], which included three large sample studies with sample sizes of n = 2320 [56], n = 2357 [79] and n = 3803 [76]. The sample size of the three longitudinal studies were n = 100 [82], n = 120 [83] and n = 617 [81]. Five studies included only female populations [73, 76, 79, 82, 94], while samples were predominantly females (64% or more) in almost half of the studies [36, 52, 53, 55, 58, 71, 72, 74, 76, 80].

### Study design

Most of the studies included in the assessment of humiliation were cross-sectional, meaning they did not examine data at multiple time-points. However, some studies [14, 86–88] included a comparison group or used retrospective data [70, 90].

Out of the twenty three studies on shame and suicidality, nineteen studies were cross-sectional or included baseline data from longitudinal studies [73, 78]. Only one study used pre-post assessment, and we included only the baseline data from that study [99]. Two studies were focused on developing or validating instruments for measuring shame, such as Multidimensional Shame related Response Inventory-MSRI [92] or Interpersonal Shame Inventory-ISI [93]). Three longitudinal studies had follow-ups of six months [82], one year [83] and two years [81].

### Measures of exposure and outcomes

Most of the studies measured humiliation as part of past stressful life events, and no validated instruments were used. Humiliation was examined as a single item categorical variable in most of the studies [68–71, 88, 90]. One study used the sub-scale of ‘adolescent stress and adversity inventory’ to measure this concept, while another three studies measured humiliation (perceived humiliation) as part of social anxiety symptoms [86, 87, 89]. In these studies, suicidality encompassed an exploration of suicidal ideation/intent, suicidal plan, suicide attempt/self-harm, and suicide. Seven studies measured suicidal ideation/intent[14, 68, 69, 71, 86, 87, 89], five studies measured self-harm/suicidal attempt [14, 68, 69, 71, 87, 89, 90] and two studies measured suicide [70, 90] in relation to humiliation. Suicidal intent or ideation was measured using varying instruments across the studies, including Pierce Suicide Intent Scale [101] used in two studies [68, 69], Suicide Ideation Questionnaire-Junior [102] in two studies [86, 89] and the Columbia Suicide Severity Rating Scale [103] in one study [87] or measured using a single categorical item (for instance, participants were asked if they had ever thought about attempting suicide during the past year) with yes/no responses [71]. Regarding suicide attempts, they were often measured as part of stressful life events, frequently utilizing a single binary yes/no item [68, 69, 71, 86, 87, 89, 90]. In one study, self-harming behaviour was assessed using a validated scale [89]. While most of these studies focused on suicide attempts as the outcome variable [68, 69, 71, 86, 87, 90], one study specifically examined self-harm [89].

Shame was measured using various validated instruments and more than 17 instruments were used across studies. The most commonly used instruments included the Test of Self-Conscious Affect-TOSCA (used in four studies) [79, 94, 95, 100] and the Objectified Body Consciousness Scale (OBCS), which included a sub-scale for measuring body shame (used in three studies) [74, 76, 83]. The Experiential Shame Scale (ESS) [75, 94] (used in two studies), Abuse Specific Shame Questionnaire (ASSQ) [73, 82] (used in two studies), and Multidimensional Shame Response Inventory (MSRI) [56, 92] were also used to measure shame. The components or dimensions of shame measured across studies included internal shame [81], external shame [91, 93], generalised shame [97], body shame [55, 74–76, 79, 83], family shame [93], shame coping–attack on self [100], shame coping—withdraw of self [100], physical or sexual abuse -related shame [73, 78, 82], shame aversion [104], “characterological shame” [75], behavioural shame [75], relationship shame [95], appearance shame [95] and performance shame [95]. Among these, the most-measured shame components were body shame, shame-proneness, and physical/sexual abuse-related shame.

Suicidal behaviours were measured using validated instruments in almost half of these studies. However, a single binary categorical item was used in four studies [56, 77, 81, 83] and a single Likert-type item [73, 82] or non-validated questionnaire/checklist [76, 78] were also used to measure the outcomes. Only two studies [94, 100] used the same instrument to measure the outcomes (for instance, Inventory of Statements about Self-Injury-ISAS), while other outcome measures varied across studies. Suicide intent or suicide ideation were measured in eleven studies [56, 73, 76, 78, 79, 81, 82, 92, 93, 95, 97] followed by non-suicidal self-injury in nine studies [74, 75, 77, 83, 91, 94, 98–100]. Only one study was specifically conducted among adolescents with suicide attempts [55].

### Prevalence of humiliation/shame

Five studies reported the prevalence of humiliation prior to subsequent self-harm or suicidal behaviours. The prevalence of humiliation was 18% [69], 28% [68, 70], 29.2% [71] and 67.1% [90] across the studies. Among these, three studies measured humiliation as part of other stressful life events, for instance, campus ragging (harassment) [71], psychological maltreatment [90] or service-related stressors [70] and the specific contribution of humiliation experiences to these stressful life events was not clearly explicated. In one study, more than 67% of adolescents with a history of psychological maltreatment (including humiliation) were involved in suicide contemplation or suicide attempts [90]. A further two studies were by the same researcher and country, with the humiliation experience among adolescents before self-harm episodes recorded at 18% [69] and 28.1% [68] respectively. Humiliation was the most experienced acute life event [14] or psychosocial stressor, and it was established as high among the adolescent group when compared across age groups [68]. Shame was measured as a continuous variable across studies, with reporting of Mean/SD only. As such, prevalence estimates were not available.

### Association between humiliation, shame and suicidality-related aspects

#### Humiliation and suicidality-related aspects

Of the ten studies pertaining to humiliation included in this review, seven have examined its association with any sub-types of suicidality, including suicidal intent/suicidal plan [14, 71, 86, 87, 89] and or self-harm/suicidal attempt [14, 71, 88, 90]. Among the five studies focusing on suicidal intent/suicidal plan, four reported this association, for instance, humiliation score was significantly higher among those with suicidal ideation [14, 71, 86, 87], although the effects were not evident in two studies after adjusting for confounders [14, 86] and one study did not include this variable in its adjusted model [71], while another study did not perform an adjusted model [87]. Of the four studies examining the association between humiliation and self-harm or suicidal attempts, two studies with good sample sizes have reported a strong positive association (for instance, OR = 3.85/OR = 4.2; p < .001)[88, 90] between these variables even after adjusting for confounders. Humiliation emerged as one of the most robust risk factors for suicidal attempts among school children [88] or the prevalence of psychological maltreatment, including humiliation, was significantly higher among children with a history of suicide attempts [90]

#### Shame and suicidality-related aspects

Out of the twenty three studies that examined the association between shame (regardless of any sub-types) and suicidality related aspects, twenty studies clearly indicated an association. For instance, higher shame scores were associated with higher levels of suicidality. This association was observed either in bivariate analysis onl [74, 77, 79, 82, 91, 92, 98] or in both bivariate and multivariate models [55, 56, 73, 76, 81, 93–95, 97, 100], and only with female [75] or male subgroups [80]. Among the three longitudinal studies, one study demonstrated the effects of shame on increasing suicidal ideation at a two-year follow-up [81], while the study of Alix et. al. (2020) failed to demonstrate this effect at six month follow-up [82]. It is noteworthy that the twelve-month longitudinal study of Duggan, Health & Hu (2015), did not treat shame as an exposure variable; instead, it examined whether adolescents with a history of non-suicidal self-injury demonstrated higher shame as compared to those without such a history [83].

*a) Shame sub-types and suicidality-related aspects*. Of the five studies examining the association between body shame and suicidality-related aspects[55, 74–76, 79], two studies reported this association in multivariate analysis [55, 76], three studies in correlational analysis [74, 75] or in an un-adjusted model [79], and only among a female population [75].

Of the three studies examining physical or sexual abuse-related shame and suicidality-related aspects, two studies reported this association in multivariate models [73, 78] or bivariate analysis [82], while this association was not evident in a six-month follow-up [82].

Of the two studies examining external shame and suicidality-related aspects, one study demonstrated this association in multivariate path analysis, and another in bivariate analysis [91]. Many other sub-types of shame were only examined in single studies.

*b) Suicidality sub-types and shame (any sub-types)*. Suicidal intent or ideation were the most reported outcomes across the studies. Among the fourteen studies examining the association between shame and suicidal ideation or intent, nine studies reported this association in multivariate models [56, 73, 76, 78, 81, 93–95, 97] and five studies reported the association in bivariate analysis [73, 74, 79, 80, 91]. One longitudinal study demonstrated this risk in a follow-up of two years [81].

Out of the nine studies on shame and non-suicidal self-injury eight studies have reported this association either in multivariate analysis [55, 75, 100] or bivariate analysis [74, 77, 91, 95, 98]. Only one study was conducted among adolescents with suicide attempts, and all the subdomains of shame were significantly higher among the attempters compared to the non-attempters [55].

*c) Shame and suicidality-related aspects (adolescent versus young adult)*. Twelve or more studies included children and adolescent populations. Among these, five studies reported an association between shame and suicidalityeven after adjusting for the covariates [55, 76, 78, 81, 97], or in bivariate analysis [79, 82, 83, 91, 98] or in an unadjusted model [79] only. Of the three longitudinal studies among adolescents, one study reported the predictors of shame on suicidal ideation at a two year follow-up [81], while others failed to demonstrate this association at a follow-up of six months [82] and 12 months [83]. Of eleven studies specific to young adults, associations were evident in six studies with adjusted models [56, 77, 93–95, 100] or bivariate analysis [74, 92] or only correlated in female [75] or male subgroups [80].

*d) Shame and suicidality-related aspects (clinical versus non-clinical populations)*. The majority of the studies on shame were conducted in non-clinical populations, involving secondary school students (6 studies), university students (10 studies), or community samples (5 studies). Among these studies, 11 reported an association between shame and suicidality in their fully adjusted models [56, 73, 76, 78, 81, 91–95, 100] and six studies reported such an association in bivariate analysis [74, 75, 77, 79, 82, 98]. Only one study was conducted in clinical settings, involving only psychiatric samples [97], which reported an association between shame and suicidal ideation. Two studies were conducted in mixed settings, including psychiatric and educational settings [55, 99], leading to mixed results regarding the association between shame and suicidality.

## Discussion

### Humiliation and suicidality-related aspects

Although humiliation has been reported as one of the most common psychosocial stressors experienced by adolescents [14, 68, 69], there is clear lack of studies in this area as only ten eligible studies were identified in our systematic search. The prevalence of humiliation among adolescents with a history of suicidal attempt ranged from 18% [69] and 29.2% [71], excluding an outlier (67.1%). However, due to small sample size, lack of comparison group (such as those with versus without a history of suicidal attempt) and high-risk of bias (based on our methodological quality assessment), it is difficult to ascertain the robustness of these results. Many studies assessed humiliation as part of stressful life events and did not provide a specific prevalence rate for humiliation, although the experience of humiliation often contributed to it. For instance, studies have reported the overall prevalence of psychological maltreatment [90] or campus ragging [71] in samples which included an item on the humiliation experience.

Regarding the association between humiliation and suicidality-related aspects, there is evidence indicating that humiliation is a risk factor for suicide attempt [88, 90], suicidal intent [71, 87], and suicidal planning [71]. However, most studies assessing this association have limitations in methodological quality, such as high-risk of bias [68–71], and issues with exposure measurement [68, 69, 71, 88, 90]. Many studies have used non-validated, single binary dichotomous items to assess humiliation [68, 69, 71, 88, 90] or post-mortem interviews with family members [70] which could result in biased or inaccurate measures.

Although three studies used validated instruments, such as a sub-scales to assess humiliation [86,87,90], the focus was on perceived humiliation only and measured as part of social anxiety symptoms. Thus, it reflects more of an anticipatory fear of humiliation rather than an actual life experience. One exception is a case control study [14] that used the stress and adversity inventory, which includes a subscale for measuring humiliation. This study had a low risk of bias and showed that humiliation (chronic difficulties) was significantly higher among adolescents with suicidal ideation and suicide attempts compared to the comparison group. However, the association was not evident when adjusting for confounders [14], which is consistent with another study in this review [86]. Therefore, while there is some evidence suggesting an association between humiliation and suicidal behaviours, the methodological limitations in the studies suggest that more research is needed to establish the robustness of this association.

To better measure the humiliation experience, we strongly recommend that future studies use specifically designed and validated instruments, such as the Humiliation Inventory [50]. As no longitudinal studies and only one case control study were identified in this area, we recommend focusing future research efforts on these study designs to establish a stronger association between humiliation and suicidal behaviours.

### Shame and suicidality-related aspects

While there was a lack of studies on the association between humiliation and suicidality among adolescent and young adults, shame and its association with various types of suicidal behaviours have been extensively examined. However, the majority of these studies were cross- sectional in nature. In this area, we have identified nineteen cross-sectional studies, three longitudinal studies, and one pre-post assessment study. Overall, more than 80% of the studies were rated as having very good methodological quality or low risk of bias.

Based on the cross-sectional studies, shame appears to be a significant risk factor for suicide ideation/suicide intent [56, 76, 78, 81, 93–95, 97], non-suicidal self-injury [74, 77, 92, 98, 100], and suicide attempts [55] among adolescents and young adults. However, since most of the included cross-sectional studies in this review were descriptive in nature (e.g. absence of a comparison group), it is difficult to establish this evidence. Generally, cross-sectional studies have limitations for making a casual inference and are unable to investigate the temporal relation between outcomes and risk factors [105]. Furthermore, the association between shame and suicidality is inconsistent in longitudinal studies. While one longitudinal study reported this association over a two-year follow-up [81], others could not demonstrate this effect [82, 83]. Nevertheless, more longitudinal studies are needed for a definitive conclusion.

Furthermore, NSSI and its related variables have often been assessed retrospectively, resulting in less precision regarding the mechanisms that contribute to the maintenance, cessation, or exacerbation of these behaviours. To achieve a more thorough understanding, employing more robust longitudinal methods like Ecological Momentary Assessment (EMA) [106] would be highly beneficial. EMA permits individuals and patients to frequently report on their experiences in real-time and real-world settings, thereby capturing changes over time and across various contexts [106]. Notably, EMA has revealed a higher prevalence of reported suicidal ideation compared to traditional retrospective self-report measure [107].

Although it is clear that an association exists between shame (in any sub-type) and suicidality, it is difficult to establish this evidence with reference to different sub-domains or dimensions of shame, except for a few, such as body shame and shame related to physical and sexual abuse. Studies have focused on various aspects of shame, with fourteen or more shame sub-types measured across studies. Among these, eleven or more subtypes of shame (e.g., internal shame, generalized shame, family shame, shame coping-attack on self, shame coping-withdraw self, shame aversion, characterological shame, behavioural shame, relationship shame, appearance shame, and performance shame) were measured in just a single study. Given this heterogeneity, a comparison of results or synthesis of evidence with reference to the above subtypes was not possible in this review.

We consider the study of specific sub-types of shame to be crucial, as they are linked with various mental health conditions, each of which can have distinct implications for suicidality. For instance, research has revealed that external shame exhibits a stronger correlation with depressive symptomatology [47] and social anxiety [108] compared to internal shame. On the other hand, internal shame might hold greater relevance than external shame in the context of generalized anxiety disorder, due to its connection with a deficit in self-reassurance [108]. Furthermore, certain studies have identified that particular sub-types of shame, such as body shame or shame related to eating, are more closely associated with eating disorders [109] in comparison to other sub-types of shame. We recommend that future studies focus on the various subtypes of shame that are less frequently addressed across studies. Another issue is the use of non-validated instruments for outcome measures of suicidal behaviours (evident in approximately half of the studies in this review), which needs to be addressed in future studies.

While the majority of the studies on shame focused on non-suicidal self-injury or suicidal ideation/intent, only one study was conducted among adolescents with suicide attempts. This trend contrasts with the studies in the area of humiliation, which extensively examined suicide attempts compared to non-suicidal self-injury. Therefore, future studies could address this knowledge gap. However, this discrepancy could also be related to the various terminologies used across the studies.

Based on this review, both humiliation and shame are potential risk factors for suicidal behaviours in adolescents and young people. Since the majority of the studies in this systematic review demonstrated this association in non-clinical samples (e.g., school children or college students), it is likely that these groups may not seek active professional help unless there is a suicide attempt or another adverse outcome. Therefore, it is crucial to educate this group about the risk of experiencing humiliation and shame on suicidal behaviours and include this information in gatekeeper or parental training as part of a comprehensive suicide prevention program for adolescents and young adults.

Regarding clinical interventions, many of the established evidence-based psychosocial methods, including Cognitive Behavioral Therapy, mindfulness, interpersonal therapy, group therapy, family therapy, expressive arts, and community-level interventions, have been found to be effective in reducing shame [110]. While research has yet to establish a strong evidence base for clinical interventions related to humiliation, empirically supported exposure therapy has been found to be beneficial for severe public humiliation [111]. Elements of dialectical behaviour therapy focused on supporting young people to understand and develop ways of tolerating shame and humiliation, enhancing interpersonal effectiveness, and building positive ways of coping with distress may also have a role in tailored interventions.

Adolescence and young adulthood are viewed as peak developmental times for the emergence of suicidality [112–114]. We suggest that the developing adolescent brain will interpret shame and humiliation in a different manner to that of an older adult, partly due to biological factors, but largely due to the primacy of, and need for social approval and peer acceptance that is so important during this stage of development. As an adolescent seeks to achieve autonomy, while trying to carve out their own identity, acceptance by peers is highly valued [115]. The effect of peer rejection on the adolescent brain has been previously explored, with neuro-imaging demonstrating increased activity in the sub-genual area of the anterior cingulate cortex, suggesting that adolescents who are very concerned with peer acceptance may be most sensitive to rejection experiences[116].

Using Erikson’s developmental framework, we can view youth suicide as emerging from the interplay of individual vulnerabilities, the disturbance of developmental tasks, and the influence of risk factors on the individual [117]. Through the conceptual lens of the Integrated Motivational-Volitional (IMV) model and Joiner’s Interpersonal Theory, it is logical to focus on the detrimental impact of humiliation and shame as they expose a pathway towards potential suicidality in vulnerable young people. Adolescents possess unique biological, cognitive, and social vulnerability factors that may shape the pre-disposing, motivational and volitional phases of the suicide process. When humiliation and shame occur, the primacy of social and peer acceptance, may distil the negative experience of these processes, and impact how a young person accesses supports. The way an adolescent responds to the process of humiliation and shame within this process will be influenced by the developing brain, stage of development, peer relations, and capacity to access nascent resilience factors.

## Conclusion

To our knowledge, this is the first study to attempt a systematic review on the impact of the lived experience of humiliation and shame on self-harm and suicidality in adolescents and young people. The dearth of research in this field of enquiry is reflective of unique challenges associated with assessments of humiliation in various clinical settings amongst adolescent and young adult populations. Humiliation and shame are potential risk factors for future suicidal ideation and self-harm among adolescents and young adults. Due to high risk of bias, reduced sample size, complexities in the objective measurement of humiliation, and lack of longitudinal studies, concerns abound in relation to the methodological quality of research on the humiliation experience in predicting adolescent suicidality. Although more studies have been carried out in relation to the experience of shame and suicidality in adolescents and young people, they were mostly descriptive and cross-sectional in nature. Only three longitudinal studies are available in this area to date, and the results of these are inconsistent. Therefore, more prospective studies are needed to establish the association between humiliation, shame and suicidal behaviours. Given the associated role of humiliation and shame in self-harm and suicidality among young people in this systematic review, we recommend that, along with gatekeeper training and education, these processes need to be assessed as part of a comprehensive and dynamic bio-psycho-social assessment when focusing on life stressors for adolescent and young adults presenting with self-harm and suicidal behaviour to emergency departments and mental health services. Focusing on the causes and influences of humiliation and shame on vulnerable young people is expected to deepen our understanding of risk and better inform tailored intervention plans.

## Supporting information

S1 Checklist PRISMA checklist. (DOCX)

S1 File Search strategy. (DOCX)
